# The Rethinking Clinical Trials (REaCT) Program. A Canadian-Led Pragmatic Trials Program: Strategies for Integrating Knowledge Users into Trial Design

**DOI:** 10.3390/curroncol28050337

**Published:** 2021-10-04

**Authors:** Deanna Saunders, Michelle Liu, Lisa Vandermeer, Mashari Jemaan Alzahrani, Brian Hutton, Mark Clemons

**Affiliations:** 1 Cancer Therapeutics Program, Ottawa Hospital Research Institute, 501 Smyth Road, Box 511, Ottawa, ON K1H 8L6, Canada; dsaunders@ohri.ca (D.S.); miliu@ohri.ca (M.L.); lvandermeer@ohri.ca (L.V.); 2 Department of Medicine, Division of Medical Oncology, The Ottawa Hospital and the University of Ottawa, 501 Smyth Road, Box 912, Ottawa, ON K1H 8L6, Canada; malzahrani@toh.ca; 3 Clinical Epidemiology Program, Ottawa Hospital Research Institute and University of Ottawa, 501 Smyth Road, Box 511, Ottawa, ON K1H 8L6, Canada; bhutton@ohri.ca

**Keywords:** breast cancer, knowledge users, patient centred outcomes, pragmatic trial

## Abstract

We reviewed patient and health care provider (HCP) surveys performed through the REaCT program. The REaCT team has performed 15 patient surveys (2298 respondents) and 13 HCP surveys (1033 respondents) that have addressed a broad range of topics in breast cancer management. Over time, the proportion of surveys distributed by paper/regular mail has fallen, with electronic distribution now the norm. For the patient surveys, the median duration of the surveys was 3 months (IQR 2.5–7 months) and the median response rate was 84% (IQR 80–91.7%). For the HCP surveys, the median survey duration was 3 months (IQR 1.75–4 months), and the median response rate, where available, was 28% (IQR 21.2–49%). The survey data have so far led to: 10 systematic reviews, 6 peer-reviewed grant applications and 19 clinical trials. Knowledge users should be an essential component of clinical research. The REaCT program has integrated surveys as a standard step of their trials process. The COVID-19 pandemic and reduced face-to-face interactions with patients in the clinic as well as the continued importance of social media highlight the need for alternative means of distributing and responding to surveys.

## 1. Introduction

**Figure 1:**
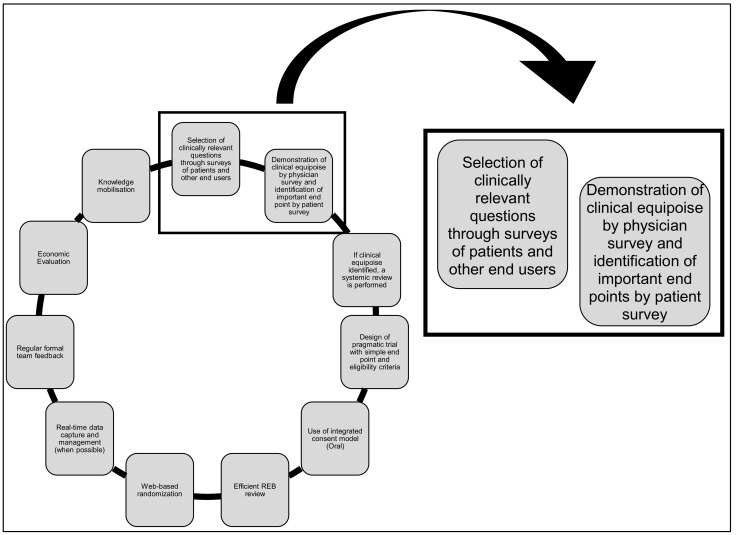
Key tenants for the REaCT Program (adapted with permission from [2,3]).

**Figure 2:**
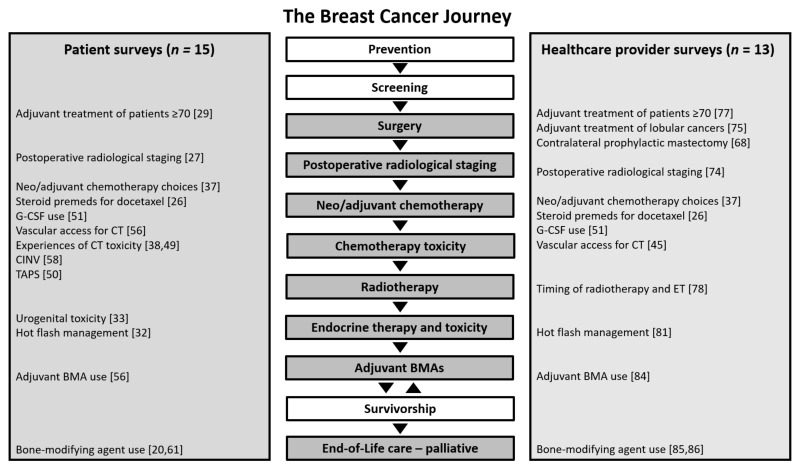
The breast cancer journey: where our surveys fit and where gaps exist. BMA = bone-modifying agent, CINV = chemotherapy-induced nausea and vomiting, CT = chemotherapy, ET = endocrine therapy, G-CSF = granulocyte colony stimulating factor, TAPS = taxane-associated pain syndrome.

There are many barriers to performing clinical trials and in recent years the number of adult cancer patients accrued to trials has steadily fallen [1]. The REthinking Clinical Trials (REaCT) Program was created with the intention of overcoming many of these barriers for comparing standard of care interventions, so that more patients could be offered participation in trials, participation would be less onerous, and results would be clinically important [2,3]. While initially developed as an initiative in Ottawa, it became increasingly clear that investigators in other centres were also interested in participating in REaCT trials as well as leading their own studies using the REaCT infrastructure. Thus over the years the program has expanded to multiple sites across Canada. The key elements of the program are shown in Figure 1, and broadly incorporate: identification of clinically relevant questions, conduct of systematic reviews of the evidence and surveys of end users, performance of pragmatic trials (using simply defined study endpoints, avoidance of superfluous data collection, use of an integrated consent model (ICM) incorporating oral consent [2,4,5], efficient Research Ethics Board (REB) approval [6], web-based randomisation in the clinic, and the use of real-time electronic data capture), economic analyses and knowledge mobilisation strategies. To date, the REaCT investigators have performed 20 randomized trials at 16 centres and has accrued over 3300 patients. The mandate of these trials has been broad, and has covered many aspects of the “cancer journey” (Figure 2) including surgery [7], pathology [8], radiology [9], device use [10,11], antiemetic support [12,13], adjuvant treatment [14], adjuvant supportive care [15] and palliative/supportive care [16]. REaCT has also performed feasibility studies to assess whether expansion to larger definitive studies would be possible [4,14,17,18].

An essential component of any trials program is obtaining feedback from potential knowledge users such as patients, health care providers (HCPs), advocacy organizations and guideline panels, i.e., those who will make use of the research results [19]. Knowledge user engagement is increasingly viewed as a fundamental part of the peer-reviewed grant process [20]. The information they can provide can include identification of areas of: variation and uncertainty in clinical practice (i.e., clinical equipoise), meaningful study endpoints, as well as raising questions of clinical importance. In addition, knowledge user feedback can provide important evidence for framing research questions and for designing clinical trials that are relevant and engaging for potential participants. Given the importance of obtaining knowledge user feedback, in this manuscript we will highlight the first 2 steps of the REaCT process (Figure 1). We present our own experience with performing surveys, including lessons learned, as well as our thoughts on how performing surveys will need to evolve with the likely irreversible reduction in in-person patient visits that has occurred as a consequence of the COVID-19 pandemic [21].

## 2. Materials and Methods

All surveys performed by the REaCT team since program inception in 2014 were reviewed as were studies performed by the team members that followed the same methodology. Where information was not available from the original publication of each survey, source documentation was sought if feasible.

### 2.1. Patient Survey Outcomes

Outcome data collected from patient surveys included patient demographics (i.e., type of cancer, stage of cancer), how potential survey participants were identified (e.g., from clinic lists), how participants were contacted for survey participation (e.g., approached by a HCP or cold-called by a study clinical research associate), how surveys were distributed to participants, and how survey responses were collected (in clinic, email, mail, various online platforms such as Microsoft Forms or the institution’s electronic medical record EMR). Where possible, information on response rates to surveys was also collected.

### 2.2. Health Care Provider Survey Outcomes

Outcome data for HCP surveys included: types of participants (e.g., surgical/medical//radiation oncologists, surgeons, RNs, APNs), how participants were identified (e.g., society listings), how participants were contacted (email, various online platforms such as Microsoft Forms), how surveys were distributed, and how survey responses were collected (in clinic, email, Microsoft Forms). Using a modified Dillman approach, each survey was sent to HCPs at least twice [22]. Where possible, information on response rates to surveys was also collected.

## 3. Results

The REaCT team members have performed and published 15 patient and 12 HCP surveys. These are outlined in Table 1 and Table 2, respectively.

### 3.1. Process for Designing Surveys

The surveys were consistently designed by a multidisciplinary team with demonstrated expertise in oncology, survey design, and methodology. Each survey was pilot tested on a limited number of patients, oncologists, advanced practice nurses and non-healthcare professionals before launch. Over time, it has become clear that repeated readings of surveys are needed to ensure that they remain clearly written with unambiguous answers. In addition, keeping surveys as short as possible to ensure compliance is essential [23].

### 3.2. Choice of Research Ethics Board (REB)

As publication of survey results is the intent of most surveys performed, we used either local REBs or, where more than one site would be accrued, we used the Ontario Cancer Research Ethics Board (OCREB). In the few examples where there was no intent to publish, no REB approval was sought. This included ad hoc surveys of colleagues in our centre asking what differences in study outcomes would be enough to drive changes in practice for the purpose of sample size application for grants. In the current review we only discus those surveys with a formal protocol that follows the REaCT program processes.

### 3.3. Use of Incentives

A significant issue with surveys is ensuring that the response rate is high enough to make the study findings truly meaningful. Some authors have proposed that survey response rates should achieve at least 60% to ensure that the validity of results is not influenced by nonresponse bias [24]. There is literature on the use of incentives (e.g., financial reward for completing the survey) as a tool for increasing response rates [25]. However, as an academic investigator-led program such incentives could be financially prohibitive to actually performing the study. In addition, any honoraria received are also taxable income that should be declared by the recipient [25]. To date, we have only had funds to offer a gift voucher (a coffee card worth $5) to those physicians who sent us an email on completion of this REB-approved survey [26].

### 3.4. Patient Surveys

Of the 15 patient surveys performed the survey topics addressed a broad range of topics including perceptions around post-operative radiological staging [27], choices of adjuvant surgery/radiotherapy and endocrine therapy in patients ≥70 [29], toxicities from endocrine therapy (hot flashes [32], urogenital side effects [33]), timing of starting endocrine therapy in patients receiving radiotherapy [78], adjuvant chemotherapy choices of chemotherapy for TNBC [37], ranking of chemotherapy toxicities for both early stage and metastatic patients [38,49], taxane-associated pain syndrome [50], use of filgrastim for primary febrile neutropenia prophylaxis for adjuvant chemotherapy [51], dosing of dexamethasone in patients receiving docetaxel [26], choice of vascular access strategy for chemotherapy administration [56], choice of endpoints for chemotherapy-induced nausea and vomiting (CINV) [58] and de-escalation of adjuvant bisphosphonates [59]. All of these surveys involved patients with breast cancer. Two surveys included patients with bone metastases, evaluating the use of bone-modifying agents (BMAs) accrued patients with breast cancer and castration resistant prostate cancer (CRPC) [61,68]. 

Of the 15 surveys performed, 5 required written consent. However, in more recent years, after working closely with local and provincial REBs all surveys used implied consent. Patients gave verbal consent to being approached for a survey and could choose to anonymously complete the survey or not. This occurred because of the increasing recognition that not all surveys required written consent and indeed the requirement for written consent could reduce the validity of study findings to reflect as broad a patient population as possible. Potential patients for surveys were often identified in the clinic (11/15), however in more recent surveys patients have also been identified and approached through their involvement in other studies [29,32] and pharmacy lists [50,68]. With the introduction of the MyChart function within the EPIC EMR patients are also now able to consent to being contacted about other studies [29,32]. Previously while most studies would accrue patients through the physician at a clinic visit it is evident that more recent studies launched since March 2021 and COVID-19 restrictions on in-person visits to the clinic have used a combination of approaches including cold calling by study CRAs [29,32,68]. However, all eligible patients were approached and presented the survey by someone in their circle of care. Traditionally, REB approval has required that paper-based copies of any survey be available for all patients for completion either in the clinic or at home and this was so for all 15 studies. However, there has been an increasing move to responses being made by; telephone (3 surveys), email (9 surveys), use of a laptop in the clinic (2 surveys), or by regular mail (4 surveys). As responses to mailed out surveys have proven to be low we are no longer offering this option. 

Using these strategies, a total of 2298 of 2624 contacted patients have responded to the 15 surveys. The median duration of the surveys was 3 months (IQR 2.5–7) and the median response rate was 84% (IQR 80–91.7%). The surveys frequently identified clinical equipoise (Table 1), and all have been either published or are currently under review [29,32]. The survey data led support to the REaCT program performing: a population-based cohort study (1), systematic reviews (10), peer-reviewed grant applications (6), review articles (3), treatment guidelines (4) and 19 clinical trials. 

### 3.5. Health Care Provider Surveys

Of the 13 HCP surveys performed, the survey topics were similar to those in the patient surveys (Table 2).These topics included: development of a decision aid for breast cancer patients considering contralateral prophylactic mastectomy [73], perceptions around post-operative radiological staging [74], management of lobular cancer [75], choices of adjuvant surgery/radiotherapy and endocrine therapy in patients aged 70 or over [77], timing of starting endocrine therapy in patients receiving radiotherapy [78], choice of chemotherapy for TNBC [37], toxicities from endocrine therapy [81], and supportive care studies for chemotherapy patients. These studies have evaluated: choice of vascular access for chemotherapy administration [83], use of growth factors with neo/adjuvant chemotherapy for breast cancer [51], dexamethasone pre-medication with docetaxel [26], as well as the de-escalation of bone-modifying agents in both the adjuvant [84] and metastatic settings [85,86]. Most studies related to the care of breast cancer patients, while the surveys evaluating bone-modifying agents in the metastatic setting [85,86] also included patients with castration resistant prostate cancer.

A broad range of HCPs were surveyed including; medical oncologists (13), radiation oncologists (9), surgical oncologists (7), oncology nurses (including advanced practice nurses (APNs) and nurse practitioners (NPs) (4), general practitioners in oncology (2), general surgeons (1), geneticists (1), urologists (1) and pharmacists (1). The method of identifying potential respondents initially came from membership listings from organizations such as the Canadian Association of Medical Oncologists (3), Canadian Society of Surgical Oncology (3), Canadian Association of General Surgeons (1), Canadian Association of Radiation Oncologists (3), Canadian Association of Nurses in Oncology (CANO) (3) and oncology nursing staff within 2 cancer centres (2). With time, these lists were used to derive a list of responsive HCPs that was used in 10 further surveys. All surveys included contacting HCPs by email and 2 also used regular mail. As these studies all received REB approval, they required a documented consent process. For the HCP surveys, completion of the survey (whether on paper or electronic) implied consent to participate in the study. 

Using these strategies, a total of 1033 of 3280 contacted HCPs responded. For 13 surveys, the median duration of surveys was 3 months (IQR 1.75–4 months) and the median response rate, where available, was 28% (IQR 21.2–49%). Similar to the patient surveys, a consequence of the 13 HCP surveys was that they frequently identified clinical equipoise (Table 2). All the surveys were published or are currently under review [77,81]. The survey data led support to: a development of a decision aid, a population-based cohort study, 6 systematic reviews, 5 peer-reviewed grant applications, 2 review articles, 2 treatment guidelines and 15 clinical trials.

## 4. Discussion

Surveys provide an important form of scientific inquiry that aim to gather reliable and unbiased data in an efficient, reasonably inexpensive, and adaptable way from a representative sample of respondents [23,24,25]. Knowledge user input through surveys is an essential part of the planning for any clinical trial. Knowledge users can provide invaluable information on such diverse issues as clinical equipoise, meaningful study endpoints, clinical importance of the question being asked, elements of study design to enhance pragmatism and improve enrollment, and willingness to participate in clinical trials (whether as a patient or as a treating physician). In this manuscript, we present the experience of the largest pragmatic oncology program that we are aware of in Canada. We also present important lessons learned regarding survey implementation thus far in the engagement of our most vital knowledge users. The lessons learned are particularly important in an era of rapid expansion of social media as well as the impact of the COVID-19 pandemic when face-to-face visits to the cancer centre are becoming less frequent and will likely remain so in the post-COVID world. 

With 15 patient surveys that received feedback from 2298 respondents, and 13 HCP surveys answered by 1033 respondents covering a broad range of mainly breast cancer-related topics, we feel we have successfully integrated surveys of knowledge users into our trials methodologies. The results of the current study show that planned collection and integration of knowledge user feedback in the Canadian health care system is feasible. These surveys have also provided information on clinical equipoise and endpoints that are important to patients. Indeed, an example was with our CINV patient survey where it was apparent that patients did not feel that the traditional endpoints used in emesis trials did not reflect the endpoints that were important to them [58]. This feedback led to a change in the design of our most recent study of CINV interventions, where nausea was made the primary endpoint [13]. Another example is the variability in filgrastim use in patients receiving chemotherapy for breast cancer [51]. This demonstration of clinical equipoise led to a successful clinical trial that showed shorter durations of filgrastim were equally effective as longer durations but with less toxicity [15]. Clearly it is therefore gratifying that our end user surveys have both directly and indirectly led to a number of important outcomes such as grant applications, systematic reviews, review papers and guidelines as well as actual clinical trials designed to answer the clinical equipoise that has been raised by end users.

Clearly as in all areas of research there are many potential limitations with performing surveys. With the need for a representative sample of respondents [23,24,25], response rates are important. Indeed, journal reviewers frequently cite low response rates as a limitation, and can also represent a barrier to publication. A growing challenge is establishing what number represents an acceptable response rate nowadays as COVID-19 has fundamentally changed the nature of clinical care with a significant reduction in face-to-face interactions between HCPs and patients. With respect to patient surveys we have explored different strategies for enhancing both the approaching of patients (for example by using pharmacy lists, as well as the MYChart function on EPIC that allows patients to consent to be approached for research endeavours). There is also an inherent bias in the types of patients approached by HCPs as they are usually under the care of investigators involved in the particular study and also rarely reflect practice across nations as a whole. Our team has also faced low response rate to telephone and mail surveys, and increasingly we are trying to perform all surveys through electronic platforms. There is also the issue that implied consent as reflected through the completion of the survey may not actually mean that the subject fully understands the objective of the study. Finally, some journals have asked us to link certain survey responses to individual patient data [59]. As surveys are typically anonymous, such post hoc analyses are not possible. With respect to HCP surveys, a challenge has been relatively low response rates. For some membership listings (e.g., CANO), we were unable to target HCPs treating a specific tumour site, meaning that response rates are at times lowered as many recipients simply do not treat that type of cancer. There is also the inherent bias of the types of HCPs who respond which is difficult to overcome. While the use of financial incentives is outlined above, these costs put this type of initiative out of reach of many investigator-non-pharmaceutical company initiated studies [25]. Another important challenge is HCP irritability at receiving unsolicited emails for survey participation. We have tried to resolve this by asking HCPs to tell us if they are not interested in receiving these emails. Finally, there exists the limitation of the surveys thus far being predominantly breast cancer-related and having a Canadian bias.

We feel end user feedback will remain an essential component of any clinical research program. Future studies are clearly needed. These could evaluate better strategies for identifying and receiving responses from as broad a range of end users as possible. Such studies could also evaluate the use of social media platforms technology. For example, for our own patients in Ottawa harnessing convenience of EPIC electronic health records to do electronic surveys may present interesting ongoing opportunities). Future studies could also potentially allow expansion of the program outside of Canada.

## 5. Conclusions

Surveys of knowledge users are an essential component of clinical research. The REaCT program has integrated surveys as a standard step of their trials process which has resulted in; grant applications, systematic reviews, review papers, guidelines and clinical trials. The COVID-19 pandemic and reduced face-to-face interactions with patients in the clinic as well as the continued importance of social media highlight the need for alternative means of distributing and responding to surveys.

## Figures and Tables

**Table 1:** Summary of patient surveys.

Reference and Year of Publication	Survey Topic	Population Surveyed	Sample Size (Response Rate)	Consent	Duration	How were Participants Identified	Methods of Approach	Methods of Completion	Pertinent Findings	Other Studies the Survey Led To
**Postoperative radiological staging**										
Simos et al., 2014 [27]	Patient perceptions regarding postoperative imaging for metastatic disease	Patients with EBC who had completed their definitive breast surgery	245/282 (87%)	Written	3 months	Eligible participants identified by their physician	Approached by their physician during a regularly scheduled visit	Paper in clinic	>80% recalled having imaging tests for distant metastases. Over half indicated they would want imaging even if the chance of detecting metastases was </=10%,	Led to a population-based cohort study [28]
**Adjuvant surgical, systemic, and radiotherapy choices in patients >70 years of age**										
Savard et al. 2021 [29]	Patient experience of the harms and benefits of radiotherapy and endocrine therapy	Patients with low risk EBC, 70 years of age or older and had been offered radiation and hormonal therapy	102/130 (78.5%)	Oral	7 months	Eligible participants identified either in outpatient clinic by their HCP or CRA if participating in other studies	Approached by their HCP in clinic or if previously transferred to Wellness program and had consented to research contact, telephoned by physicians or CRA	Paper in clinic/Mail/ Emailed web-based survey/Telephone	Most patient received radiation and endocrine therapy and that have minimal or no impact on their quality of life. Most respondents preferred radiation over endocrine therapy if they had to choose between the two treatment modalities.	Led to systematic review [30] and pilot clinical trial [31]
**Supportive care—endocrine therapy**										
Cole et al., 2021 [32]	Patient experience of hot flashes and efficacy of prior treatments	Patients with EBC who were experiencing hot flashes	373/448 (83%)	Oral	9 months	Eligible participants identified either in outpatient clinic by HPC, or by CRAs if participating in other studies	Approached by their HCP in clinic or if previously trasferred to Wellness program and had consented to research contact, telephoned by physicians or CRA	Paper in clinic/Mail/ Emailed web-based survey /Telephone	Most patients with VMS did not feel the issue was adequately acknowledged or addressed. Patients wanted better and more personalized approaches to VMS management.	Led to grant application
Chin et al., 2009 [33] *	Prevalence of urogenital symptoms in postmenopausal patients with BC receiving endocrine therapy	Postmenopausal women receiving endocrine therapy for EBC or metastatic BC	251 (response rate N/A)	Written	3 months	Eligible participants were identified by their physician	Eligible participants were approached by their physician during a regularly scheduled visit	Paper in clinic	Urogenital side effects reported by 63% of patients. Less than one third of patients had used some form of treatment for these symptoms.	Led to review article [34], systematic review [35] and clinical trial [36]
**Adjuvant chemotherapy choices for EBC and metastatic breast cancer**										
Jacobs et al., 2017 [37]	Adjuvant CT choices for EBC. Willlingness to participate in trials. Thoughts on the ICM	Patients with EBC and all receptor types treated with neo/adjuvant CT	74 (response rate N/A)	Oral	4 months	Eligible participants identified by their physician	Participants approached by their physician during a regularly scheduled visit	Paper in clinic/Take home	Most respondents willing to participate in trials to determine optimal CT regimens. Respondents interested in studies to minimize side effects, even if this means longer duration of treatment. Most respondents willing to enter clinical trials if administrative processes around trial entry were streamlined.	Led to a clinical trial [14]
Beusterien et al., 2014 [38]	Conjoint analysis to assess BC patient preferences for CT side effects	Female patients with BC receiving CT for any stage of breast cancer	102 (response rate N/A)	Written	7 months	Eligible participants identified by their physician	Participants approached by their physician during a regularly scheduled visit	Web-based (laptop in the clinic or at home)	Identified relative preferences for side effects from the patient perspective. Patients willing to make trade-offs between side effects and different routes and schedules of treatment.	Led to systematic review [39,40,41,42], reviews [43,44] and clinical studies [12,13,45,46,47,48]
Kuchuk et al., 2013 [49]	To obtain utility weights from patients with BC for common side effects of CT	Female patients with BC receiving CT for any stage of breast cancer	69 (response rate N/A)	Written	7 months	Eligible participants identified by their physician	Participants approached by their physician during a regularly scheduled visit	Web-based (laptop in the clinc or at home)	The least preferred side effects of CT were: nausea/vomiting, diarrhea, neuropathy. Survival was more important than slowing cancer growth and maintaining quality of life.	Led to systematic review [39,40,41,42], reviews [43,44] and clinical studies [12,13,45,46,47,48]
Saibil et al., 2010 [50] *	Incidence of taxane-induced pain and distress	Patients with EBC treated with anthracycline-taxane CT	82 (response rate N/A)	Written	N/A	Eligible participants identified through pharmacy and hospital records	Participants approached by their physician during a regularly scheduled visit	Interview	Distressing taxane-induced pain was common. Myalgias and arthralgias were major component of distress experienced. Pain required narcot ics in 43% of patients.	Led to systematic reviews [39,40,41], guidelines , clinical study [47,45]
**Supportive care—adjuvant chemotherapy**										
Hilton et al., 2018 [51]	Filgrastim use in patients receiving CT	Patients with EBC treated with CT	95/97 (98%)	Oral	3 months	Eligible participants identified by their physician	Participants approached by their physician during a regularly scheduled visit	Paper in clinic/ Emailed web-based survey	Patients willing to participate in clinical trials to evaluate optimal duration of G-CSF. Respondent preference was for prophylaxis with antibiotics over G-CSF, if there is no difference between the two.	Led to systemaitic reviews [52,53], clinicl trials [4,15,17,54]
Jacobs et al., 2015 [26]	Optimisation of steroid prophylaxis schedules for patients with BC receiving docetaxel CT	Patients with EBC treated with docetaxel CT	72/87 (82.3%)	N/A	N/A	Eligible participants identified by their physician	Participants approached by their physician during a regularly scheduled visit	Paper in clinic	A single steroid protocol for pre- and post-medication prophylaxis is required. A single protocol for post-medications required when pre-medication not taken as prescribed.	Led to a clinical trial [55]
LeVasseur et al., 2018 [56]	Determine patient experience of vascular access (peripheral access, PICC and PORT) for administering CT	Patients with EBC who had received anthracycline-cyclophosphamide-based CT	187/200 (93.5%)	Oral	3 months	Eligible participants identified by their physician	Participants approached by their physician during a regularly scheduled visit	Paper in clinic	Respondents report being satisfied with the vascular access used for their treatment. Perceived risk factors for lymphedema were variable and are not evidence-based.	Led to systematic review [57] and clinical trials [10,11]
Hernandez Torres et al., 2015 [58]	Patient experiences of CINV and perceptions of different CINV assessment tools	Patients with EBC who had received anthracycline-cyclophosphamide-based CT	168/201 (83.6%)	Oral	7 months	Eligible participants identified by their physician	Participants approached by their physician during a regularly scheduled visit	Paper in clinic/Mail/ Telephone	Respondents strongly favor a CINV endpoint that includes the absence of both nausea and vomiting. Respondents experience with CINV is underestimated when nausea is not included in composite end points.	Led to systematic review [42], review [43,44], 2 grant applications and clinical trials [12,13,48]
**Adjuvant bisphosphonate therapy**										
McGee et al., 2021 [59]	Patient experiences adjuvant BP use and future trial designs for adjuvant BPs	Patients with EBC who had either completed or were currently receiving adjuvant BPs	164/255 (64.3%)	Oral	2 months	Eligible participants identified by their physician	Participants approached by their physician during a regularly scheduled visit	Paper in clinic/Mail/ Emailed web-based survey /Telephone	More than 50% of respondents were interested in a BP de-escalation trial	Led to guidelines [60], pilot study of different dosing durations [18]
**Palliative/Supportive Care: bone-modifying agents (BMAs)**										
Hutton et al., 2013 [61]	Patient experiences of palliative BMA use and future trials of treatment de-escalation	Patients receiving BMAs for metastatic prostate or BC	141 patients, 76 (53.9%) with prostate cancer and 65 (46.1%) with BC	N/A	3 months	Eligible participants identified by their physician	Participants approached by their physician during a regularly scheduled visit	Paper in clinic/Take home/Web-based in clinic	Different BMAs used in prostate and BC. Perceptions of the goals of therapy similar. Patients were interested in participating in trials of de-escalated therapy.	Led to systematic review [62,63] guidelines [64,65] and clinical trials [16,66,67]
AlZahrani, 2021 [68]	Patient experiences of palliative BMA use and future trials de-escalation after 2 years of treatment	Patients receiving BMAs for metastatic prostate or BC	172/220 (78.2%)	Oral	2 months	Eligible participants identified by their physician and from pharmacy lists	Participants approached by their physician during a regularly scheduled visit or cold calling by CRA	Paper in clinic/Mail/ Emailed web-based survey /Telephone	Respondents interested in trials of de-escalated therapy. Quality of life is an important clinical endpoint.	Led to review paper [69], systematic reviews [70] and clinical trials [16,71,72]

* While started before REaCT was formally established, the study follows the REaCT mandate.

**Table 2:** Summary of health care provider surveys.

Reference and Year of Publication	Survey Topic	Population Surveyed	Sample Size (Response Rate)	Duration	How were Participants Identified	Methods of Approach	Methods of Completion	Summary of Pertinent Findings	Other Studies the Survey Led To
**Contralateral prophylactic mastectomy**									
Squires et al., 2019 [73]	Development of a patient decision aid for contralateral prophylactic mastectomy (cpm)	Medical/ surgical/ radiation oncologists, plastic surgeons, general surgeons, oncology nurses, geneticists	39 (response rate N/A)	N/A	Master lists were compiled using publicly available information in databases	Invited by email	Emailed web-based survey	The cpm patient decision aid can be used by clinicians in consultation with women who have unilateral BC to enhance evidence-informed and shared decision-making with respect to undergoing cpm	N/A
**Postoperative radiological staging**									
Simos et al., 2015 [74]	Physician perceptions around radiological imaging of patients with newly diagnosed BC	Canadian breast cancer surgical, radiation, and medical oncologists	173/665 (26%)	4 months	Email lists from Canadian Society of Surgical Oncology, Canadian Association of General Surgeons, Canadian Association of Radiation Oncologists and Canadian Association of Medical Oncologists	Invited by email	Emailed web-based survey	The majority of physicians treating BC patients are aware of and generally agree that guidelines pertaining to staging imaging for EBC are reflective of evidence. Despite this, adherence is variable.	Led to a population-based cohort study [28]
**Adjuvant surgical, systemic, and radiotherapy choices for breast cancer patients**									
Jacobs et al., 2015 [75]	Management approaches, evidence supporting practice, and future research needs for management of invasive lobular carcinoma	Canadian breast cancer surgical, radiation, and medical oncologists	88/428 (20.6%)	N/A	Canadian Society of Surgical Oncology, Canadian Association of General Surgeons, Canadian Association of Radiation Oncologists and Canadian Association of Medical Oncologists	Invited by email	Emailed web-based survey	Variation exists in physicians’ beliefs around the quality of evidence for the management of invasive lobular carcinoma	Led to a review [76]
AlZahrani et al. 2021 [77]	Adjuvant management strategies for older patients with low risk HR positive early stage breast cancer	Canadian breast cancer surgical, radiation, and medical oncologists	50/242 (21%)	3 months	Collection of publicly available email addresses used by the research team in previous surveys	Invited by email	Emailed web-based survey	There is interest in trials of different adjuvant strategies in regard of radiation and endocrine therapy	Led to systematic review [30] and pilot clinical trial [31]
McGee et al., 2019 [78]	Physician recommendations for the timing of starting endocrine therapy either before, concurrent with, or sequential to radiotherapy for patients with EBC	Canadian breast cancer radiation and medical oncologists	65/220 (30%)	3 months	Collection of publicly available email addresses used by the research team in previous surveys	Invited by email	Emailed web-based survey /Paper	Decisions around the timing of endocrine therapy and radiotherapy are largely made based on physicians’ personal choices.	Led to a systematic review [79] and a clinical trial [80]
Jacobs et al., 2017 [37]	Physician preferred CT for early stage TNBC and clinical trial strategies.	Medical oncologists	41/84(48.8%)	3 months	Medical oncologists who had responded to previous practice-based surveys	Invited by email	Emailed web-based survey	Optimization of chemotherapy for TNBC is an important and unmet clinical need. The majority of medical oncologists are interested in entering trials to optimise CT choices	Led to a clinical trial [14]
**Supportive care—endocrine therapy**									
Cole et al., 2021 [81]	HCP recommendations for management of hot flashes in patients with EBC	Canadian surgical, radiation, and medical oncologists, general practitioners in oncology, nurse practitioners, oncology nurses specializing in BC	Physicians: 36/212 (17%) Nurses: 29 (response rate N/A)	4 months	Collection of publicly available email addresses used by the research team in previous surveys. Canadian Association of Nurses in Oncology (CANO) membership email pool	Invited by email	Emailed web-based survey	54% of HCPs reported being confident in managing these symptoms. The most commonly recommended intervention was antidepressants. HCPs desire optimal treatment strategies. HCPs lack comfort and experience in prescribing complementary/ alternative medicine therapies.	Led to systematic review [82], grant application
**Supportive care—adjuvant chemotherapy**									
LeVasseur et al., 2018 [83]	Determine current access practices, perceptions of complications with vascular access (peripheral access, PICC and PORT) for administering CT. Evaluated perceived risk factors for lymphedema	Canadian oncologists and oncology nurses responsible for the care of breast cancer patients	Physicians: 25/27 (93%) Nurses: 57 (response rate N/A)	4 months	Collection of publicly available email addresses used by the research team in previous surveys. Nurses were approached by their respective nurse managers.	Invited by email/ Approached by manager	Emailed web-based survey /Paper	Type of venous access used for administering CT treatment varies significantly, as do perceptions about the risks of vascular device use. Many ”urban legends” about risk factors for lymphedema persist amongst HCPs	Led to systematic review [57] and clinical trials [10,11]
Hilton et al., 2018 [51]	Determine current practices for granulocyte colony-stimulating factor (G-CSF) use for CT in EBC.	Canadian oncologists involved in the treatment of breast cancer patients	38/50 (76%)	3 months	Collection of publicly available email addresses used by the research team in previous surveys	Invited by email	Emailed web-based survey	Significant variability in practice exists. Definitive studies are required to standardize and improve care.	Led to systematic reviews [52,53], clinical trials [4,6,15,17]
Jacobs et al., 2015 [26]	Optimisation of steroid prophylaxis schedules for patients with BC receiving docetaxel CT	Oncology nurses, oncology pharmacists and medical oncologists	184/698 (26.4%)	N/A	Members of Canadian oncology societies, and oncology nurses working at cancer centres.	Invited by email/ Nurses approached at cancer centres	Emailed web-based survey/Paper	A single steroid protocol for pre- and post-medication prophylaxis is required. A single protocol for post-medications is required when pre-medication not taken as prescribed.	Led to a clinic trial [55]
**Adjuvant bisphosphonate therapy**									
McGee et al., 2021 [84]	Determine real world practice patterns of adjuvant BMA use in treatment of patients with EBC and to determine interest in clinical trials of alternative strategies for BMA administration.	Canadian oncologists treating patients with EBC	53/127 (41.7%)	1 month	Collection of publicly available email addresses used by the research team in previous surveys	Invited by email	Emailed web-based survey	Questions around optimal use of adjuvant BMAs still exist. There is interest in performing trials of de-escalation of these agents.	Led to pilot study of different dosing durations [18]
**Palliative/Supportive Care: bone-modifying agents (BMAs)**									
Hutton et al., 2013 [85]	Assess current clinical practice regarding the use of BMAs in patients with metastatic breast and prostate cancer.	Survey respondents were medical oncologists (71.1%), radiation oncologists (21.1%) and urologists (7.8%)	90/193 (49%)	N/A	Participants from previous national annual meetings related to this study	Invited by email	Emailed web-based survey	Significant areas of clinical equipoise with respect to use of BMAs exist. Physicians are interested in de-escalated therapy for breast and prostate cancer patients.	Led to systematic review [62,63] guidelines [64,65] and clinical trials [16,66,67]
AlZahrani et al., 2021 [86]	Identify current practices, as well as perceptions around long-term BMA use, BMA de-escalation, and further BMA de-escalation after 2 years of use.	Canadian oncologists treating BC or CRPC	65/295 (22%)	4 weeks	Collection of publicly available email addresses used by the research team in previous surveys	Invited by email	Emailed web-based survey	Most physicians are de-escalating BMAs. There is equipoise re: continuing BMA beyond 2 years. Survey gave favoured study endpoints for future prospective studies.	Led to clinical trials [16,72,87,88]
